# Chicago Public Health Department Social Media Communications on Twitter During the COVID-19 Pandemic and the Mpox Epidemic: Cross-Sectional Content Analysis

**DOI:** 10.2196/68200

**Published:** 2025-07-18

**Authors:** Matthew R Boyce, Margot Gordon, Rachael Piltch-Loeb, Rebecca Katz

**Affiliations:** 1 Department of Health Policy and Management, Texas A&M School of Public Health, 212 Adriance Lab Road, College Station, TX, 77843, United States, 1 9794369466; 2 Center for Global Health Science & Security, Georgetown University, Washington, DC, United States; 3 Department of Environmental, Occupational, and Geospatial Health Sciences, CUNY Graduate School of Public Health & Health Policy, New York, NY, United States

**Keywords:** content analysis, COVID-19, infodemic, mpox, public health, risk communication, social media, urban health

## Abstract

**Background:**

Protecting public health depends on the effective communication of health-related information to the public, especially during public health emergencies. Health communication campaigns traditionally relied on mass media outlets but increasingly incorporate social media platforms. This paper presents a content analysis of original communications posted to X (formerly Twitter) by the Chicago Department of Public Health (CDPH) from May 1, 2022, to April 30, 2023, a time characterized by the concurrent COVID-19 pandemic and mpox epidemic public health emergencies.

**Objective:**

This paper aims to investigate: (1) what information was being discussed by CDPH, (2) how information was presented, (3) the nature of communications, and (4) the impact of communication attributes on engagement. Secondary objectives included investigating the correlation between mpox cases and mpox-related communications and using a bioethical risk communication framework to characterize the intent of mpox-related communications.

**Methods:**

Original communications posted by the CDPH from May 1, 2022, to April 30, 2023, were collected. Communication attributes including the date and time of posting, the communication text, accompanying media, text in image-based accompanying media, and the language of the text were extracted at the time of collection. A total of 2 researchers independently reviewed the communications using a coding schema that was developed to codify the health topics and the bioethical framework to codify the intent of mpox-related communications. Percent agreement and Cohen kappa were used to establish intercoder reliability. Negative binomial regressions were used to investigate the impact of attributes on public engagement. Spearman rank correlation coefficients were used to measure the strength and direction of the correlation between the weekly number of mpox cases and the number of weekly mpox-related communications.

**Results:**

A total of 1105 original communications were posted, a majority of which discussed communicable diseases (n=539, 51.8%), were posted in English (n=801, 72.5%), during the standard workday (n=1003, 90.8%), and with additional media (n=839, 75.9%). All communications were proactive in nature, and none directly responded to other accounts. Regression analysis suggested that communications posted during the workday (event rate ratio [ERR]=1.25) and those with images (ERR=2.59) or videos (ERR=2.40) received significantly higher levels of engagement, as did those discussing maternal and child health (ERR=2.35), mental health (ERR=1.48), and substance use (ERR=1.61). Communications discussing communicable diseases were not among the health topics with higher levels of engagement. Communications posted exclusively in Spanish received significantly lower levels of engagement (ERR=0.67). In addition, mpox-related communications were positively correlated with reported mpox cases at a significant level, and most mpox-related communications sought to inform the public (n=60, 60.6%), as opposed to influence behavior (n=39, 39.4%).

**Conclusions:**

Social media platforms can represent valuable tools for risk communication during public health emergencies but should supplement other dissemination vehicles that may be more appropriate for communicating nuanced information, achieving behavior change, and reaching certain demographic groups.

## Introduction

The protection of public health depends on the effective communication of health-related information to the public. As such, public health communication represents a key mechanism by which officials engage the public to inform and educate, promote action, and garner support for public health interventions.

This is particularly true in times of crisis or emergency. Public health emergency risk communication represents an essential function of public health agencies and a critical responsibility of officials during emergency responses [1-3]. During a public health emergency, individuals use numerous information sources. These sources include social networks (ie, family, friends, and medical professionals), traditional mass media (eg, print news, television, and radio), and digital media (eg, online outlets and social media). Historically, health communication campaigns have relied on traditional mass media sources for information dissemination [4]. However, with the advent and increasingly ubiquitous nature of social media, message delivery strategies have become more diverse and increasingly incorporate social media platforms [4-6].

The social media platform X (formerly known as Twitter) is widely used in the United States. Since 2015, greater than 1 in 5 American adults report that they use X, and the respective share of Americans who report using the platform remained statistically unchanged from 2019 through at least 2023 [7]. The use of X among local health departments (LHDs) in the United States mirrors this trend. In 2010, 13% of LHDs reported using X, but this number had grown to 34% by 2022—including 26% reportedly using the social media platform during emergency responses [8].

Public health officials have primarily used X to inform the public about a variety of health topics including diabetes, tobacco, routine immunizations, sex education, and prenatal health [9-12]. However, officials have also used social media platforms, including X, to perform a variety of critical functions during emergencies. These include informing the public about emergency response efforts, directing attention to resources and intervention sites, combating infodemics, mobilizing community partnerships, understanding public perceptions and opinion, and collecting surveillance data [13-16].

As the COVID-19 pandemic began to wane in 2022, an international outbreak of mpox (ie, formerly monkeypox) began in May 2022, declared a Public Health Emergency of International Concern in July 2022, and caused widespread apprehensions about the potential for another viral pandemic [17]. This emergency declaration remained in effect until May 2023 when the Director General of the World Health Organization announced that the international emergencies for both the COVID-19 pandemic and mpox epidemic were over [18].

While a rapidly expanding body of research explores the intersection of social media and these infectious disease outbreaks, much of this focuses on the public sentiments expressed as well as the rapid spread of misinformation, disinformation, and malinformation on social media. Conversely, relatively little literature has investigated how health authorities used social media platforms during these public health emergencies. This paper works to address this gap and presents a case study of original communications posted to X by the Chicago Department of Public Health (CDPH) from May 2022 to April 2023—a period characterized by the concurrent public health emergencies of the COVID-19 pandemic and mpox epidemic. More concretely, this paper explores the content and intent of CDPH communications on X and asks four principal research questions: (1) what information was being discussed by the CDPH, (2) how was information presented to the public, (3) what was the nature of social media communications, and (4) how did communication attributes public engagement? It also examines the correlation between reported mpox cases and mpox-related communications and characterizes the intent of mpox-related messaging.

## Methods

### Data Collection

Original CDPH communications posted to X were manually collected from May 1, 2022, to April 30, 2023. At the time of collection, each communication was assigned an identification number, and several attributes were extracted as data including the date and time (local time) communications were posted, the text included in the communication (ie, the primary text), accompanying media (ie, images or videos), the text contained in image-based accompanying media (ie, the secondary text), the language of the primary and secondary text, and the nature of the communication (ie, if the communication was proactively communicating or reactively responding to content posted by other accounts). The hyperlink to the communication and the date the communication was last accessed were also documented.

On December 15, 2022, X implemented a new policy allowing users to see the number of times a communication was viewed for all communications posted after this date. The number of views received by a communication was collected following this policy change as a means of quantifying public engagement. These data were collected on May 31, 2023, to ensure that at least a month had passed since a communication was posted.

### Data Coding

A coding schema was developed to codify the primary health topic and subtopics discussed in communications (see Multimedia Appendix 1). Primary health topics included: communicable disease, environmental health, maternal and child health, mental health, noncommunicable disease, public safety and violence, substance use, multiple health topics, other health topics, and other non-health topics. A list of subtopics was iteratively developed to further classify communications.

A total of 2 researchers independently reviewed the communications and coded the primary health topic and subtopics. If a communication was coded as relating to multiple health topics or multiple subtopics, the topics were noted by coders. Once coding was complete, results were reviewed, and 2 measurements of intercoder reliability—the percent agreement and Cohen kappa—were calculated to assess intercoder reliability [19,20]. Interpretation of Cohen kappa relied on the thresholds proposed by McHugh, in which a kappa statistic of 0.00‐0.20 indicates no agreement, 0.21‐0.39 indicates minimal agreement, 0.40‐0.59 indicates weak agreement, 0.60‐0.79 indicates moderate agreement, 0.80‐0.90 indicates strong agreement, and 0.90‐1.00 indicates almost perfect agreement [20]. Discrepant coding results were discussed by researchers until a consensus was reached.

### Data Analysis

Summary statistics were used to describe the data before regression models were used to examine the relationship between engagement and communication attributes. More specifically, the number of views received by a communication was used to quantify engagement, while the attributes investigated included the presence of additional media (image or video), the language (English, Spanish, or bilingual), the time a communication was posted, and the primary health topic. Negative binomial regressions were used because the number of views (ie, the dependent variable) was an over-dispersed count outcome. The respective referent groups for each attribute were communications with no additional media, English language communications, communications posted outside of the standard workday (9 AM to 4:59 PM), and other health topics. The threshold for statistical significance was set at an alpha value of .05 (*P*<.05). Data analyses were conducted using Stata BE (version 17; StataCorp) in December 2023.

### Analysis of Mpox-Related Communications

Communications that were coded as primarily relating to communicable disease and with an mpox subtopic underwent additional review to codify communication intent. The coding schema used was grounded in a bioethical framework for classifying the intent of communications during a public health emergency [21].

**Figure 1.**
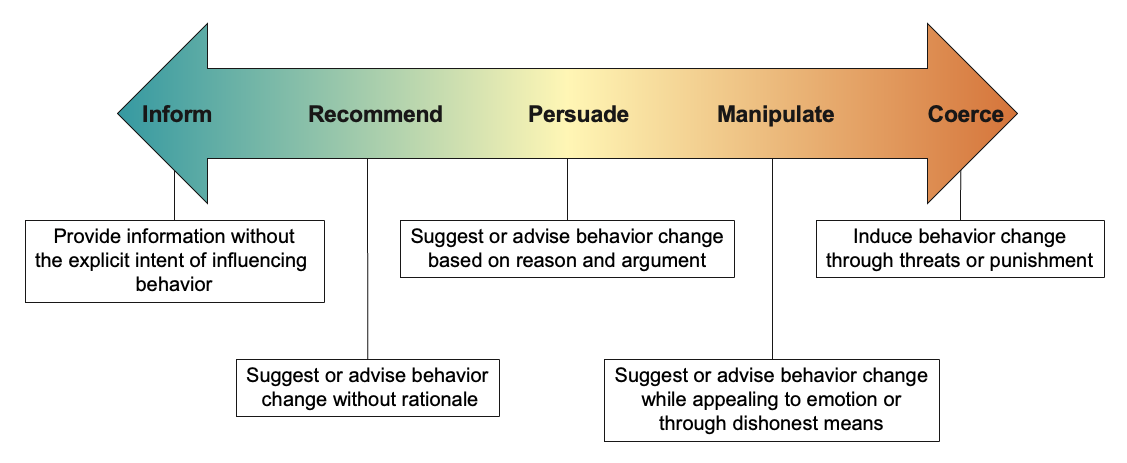
Bioethical framework for classifying the intent of communications during a public health emergency; adapted from work by Oxman et al [21].

The framework proposes a continuum that spans informing the public to coercing behavior change (see Figure 1). More specifically, the framework suggests that communications can inform the public by providing information without the explicit intent of influencing behavior, or can seek to influence behavior by making recommendations without the provision of rationale (ie, recommend), making recommendations based on reason and argument (ie, persuade), by appealing to emotion or through dishonest means (ie, manipulate), or by threats or punishment (ie, coerce). This framework was chosen over other public health communication frameworks because it was designed specifically for public health communications in the context of epidemics and pandemics, which differ from public health communications in other contexts not characterized by emergencies [21].

Weekly reported mpox cases were collected from the Chicago Department of Public Health’s website for inclusion in a correlational analysis investigating the relationship between mpox-related communications and reported mpox cases. This analysis considered a timeframe of 4 weeks before the first mpox case was reported and 4 weeks of no reported cases (ie, weeks 1‐43). Spearman rank correlation coefficients were used to measure the strength and direction of the correlation between the weekly number of reported mpox cases and the number of weekly mpox-related communications [22,23]. Correlation coefficients were calculated for the number of reported mpox cases and the number of mpox-related communications in the same week, and when constructing 1- and 2-week communication lags to examine the strength of correlations between the number of reported mpox cases and the number of mpox-related communications in the following weeks.

### Ethical Considerations

This research did not involve human subjects and involved the analysis of publicly available data. Accordingly, no ethical review was sought for this study, and data were not required to be anonymized or deidentified.

## Results

### Principal Results

A total of 1105 original communications were posted by the CDPH from May 1, 2022, to April 30, 2023 (see Table 1). On average, the CDPH posted 21.2 (SD 7.0) communications per week, with a median of 21 (IQR 15.7‐26.0) communications posted per week. The maximum number of communications posted per week was 39 and the minimum number of communications posted per week was 9. View count data were available for 376 of the communications. View counts ranged from 148‐44,900 views, and the average views per communication was 2390.9 (SD 3795.4).

**Table 1.** Descriptive statistics for attributes of Chicago Department of Public Health X communications from May 1, 2022, to April 30, 2023 (N=1105).

Variable	Value
Weekly communications	
Mean (SD)	21.2 (7.0)
Median (IQR)	21 (15.7‐26.0)
Views (n=376)	
Mean (SD)	2390.9 (3795.4)
Median (IQR	1146 (913.5‐1781.0)
Language, n (%)	
English	801 (72.5)
Spanish	67 (6.1)
Bilingual	237 (21.4)
Media, n (%)	
No media	266 (24.1)
1 image	421 (38.1)
2 images	202 (18.3)
3 images	21 (1.9)
4 images	37 (3.3)
Video	158 (14.3)
Time (local time), n (%)	
7 AM to 7:59 AM	5 (0.4)
8 AM to 8:59 AM	26 (2.3)
9 AM to 9:59 AM	184 (16.6)
10 AM to 10:59 AM	110 (9.9)
11 AM to 11:59 AM	158 (14.3)
12 PM to 12:59 PM	125 (11.3)
1 PM to 1:59 PM	114 (10.3)
2 PM to 2:59 PM	97 (8.8)
3 PM to 3:59 PM	103 (9.3)
4 PM to 4:59 PM	112 (10.1)
5 PM to 5:59 PM	43 (3.9)
6 PM to 6:59 PM	12 (1.1)
7 PM to 7:59 PM	15 (1.4)
8 PM to 8:59 PM	1 (0.1)
Nature, n (%)	
Proactive	1105 (100)
Reactive	0 (0)

English and Spanish were the only languages used in communications. Out of the 1105 communications, 801 communications (72.5%) were posted in English, 67 (6.1%) were posted in Spanish, and 237 (21.4%) were bilingual and contained both English and Spanish text. A total of 839 communications (75.9%) contained additional media—421 (38.1%) included 1 image, 202 (38.1%) included 2 images, 21 (1.9%) included 3 images, 37 (3.3%) included 4 images, and 158 (14.3%) included a video. The earliest time a communication was posted was at 7 AM, the latest was at 8:48 PM, and 1003 of the communications (90.8%) were posted during the standard workday (ie, 9 AM to 4:59 PM). All communications were proactive in nature, and none were reactive.

**Figure 2.**
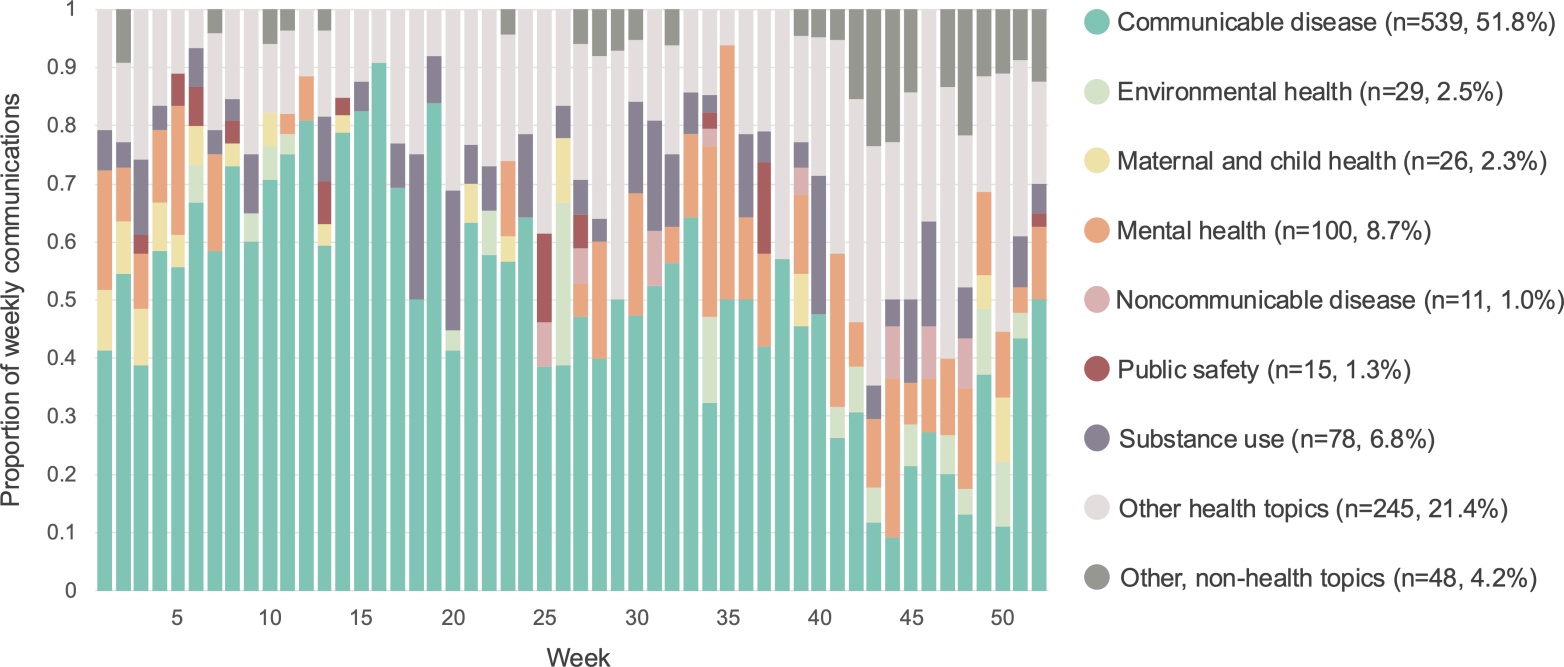
Proportion of health topics discussed by the Chicago Department of Public Health communications (May 1, 2022, to April 30, 2023).

The schema used to codify the topic of communications was robust. The coders agreed on 990 of the 1105 communications, yielding an 89.6% agreement rate and a Cohen kappa of 0.84, which is indicative of strong levels of agreement [24,25]. A total of 539 of the communications (51.8%) discussed communicable diseases, 245 (21.4%) discussed other health topics, 100 (8.7%) discussed mental health, 78 (6.8%) discussed substance use, 48 (4.2%) discussed other non-health topics, 29 (2.5%) discussed environmental health, 26 (2.3%) discussed maternal and child health, 15 (1.3%) discussed public safety and violence, and 11 (1.0%) discussed noncommunicable diseases (see Figure 2).

**Figure 3.**
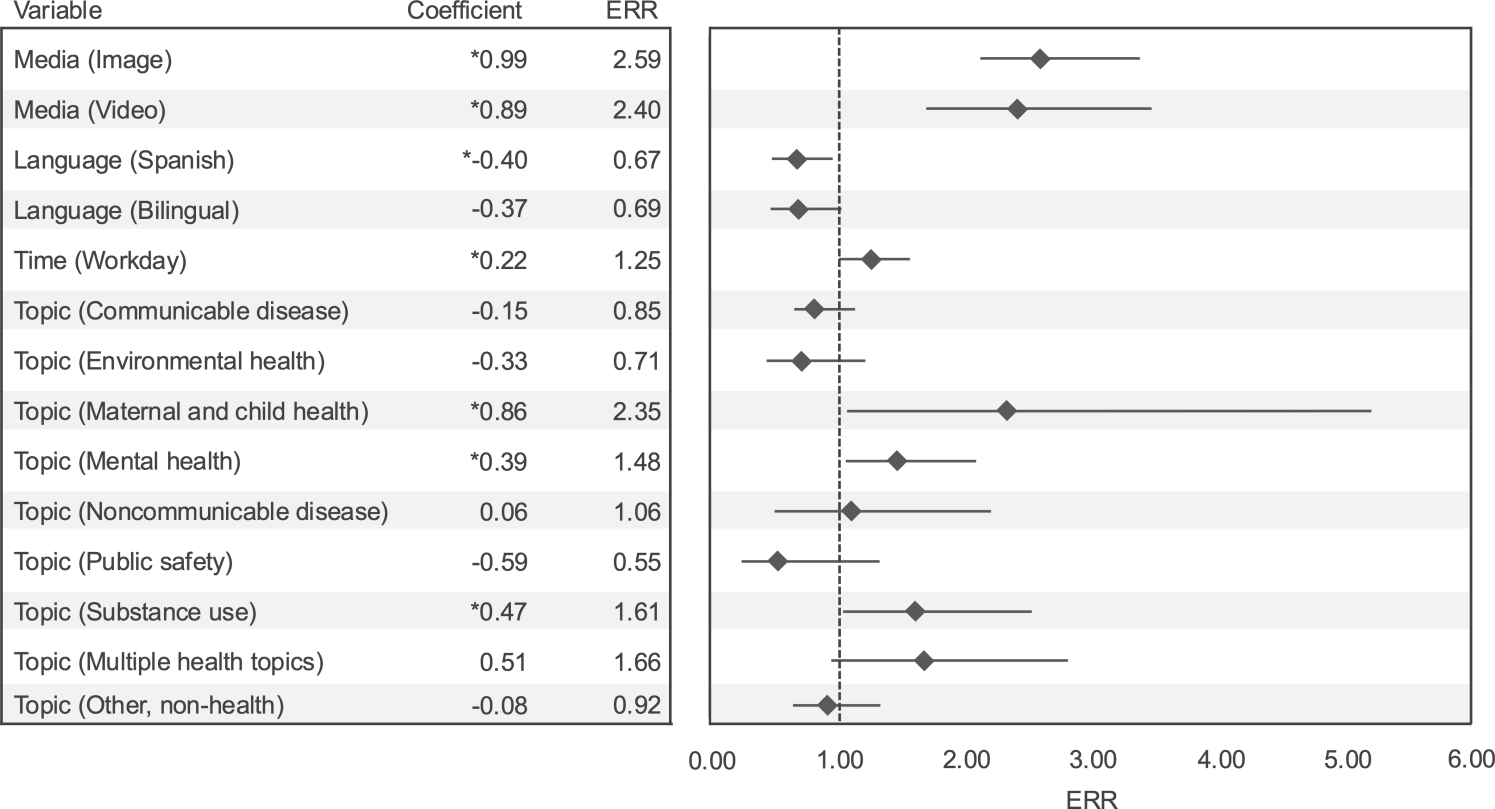
Negative binomial regression coefficients and ERRs for the relationship between dependent variables of interest and communication views. ERR: event rate ratio. * *P*<.05

Negative binomial regressions suggested several factors were significantly associated with the number of views a communication received (see Figure 3). Compared to communications that did not include additional media, the view rate for communications with images was 2.59 times higher (*P*<.001), and the view rate for communications with videos was 2.40 times higher (*P*<.001). Communications posted only in Spanish received significantly lower engagement, as the view rate for communications in Spanish was 0.67 times the rate for communications posted in English (*P*=.03). Communications posted during the workday received significantly higher engagement compared to those posted outside of the standard workday, as the view rate for communications posted during the workday was 1.25 times the rate for communications posted outside of the workday (*P*=.045). Communications discussing maternal and child health, mental health, and substance use received significantly higher levels of engagement, with respective view rates of 2.35 (*P*=.009), 1.48 (*P*<.001), and 1.61 (*P*=.001) times higher than the rate for communications discussing other health topics.

### Mpox-Related Communications Results

**Figure 4.**
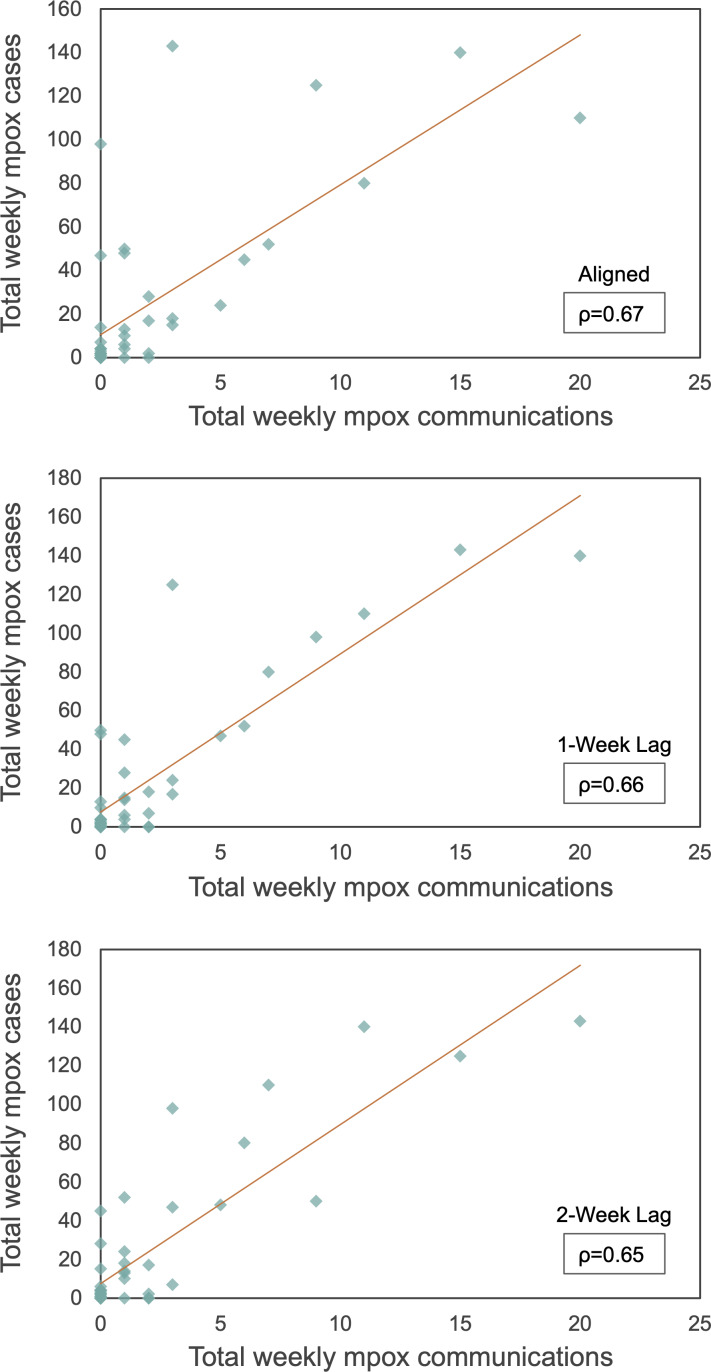
Scatterplots of weekly reported mpox cases and weekly mpox-related communications (weeks 1‐43) aligned temporally, with communications lagged 1 week and with communications lagged 2 weeks.

From May 1, 2022, to April 30, 2023, a total of 1131 mpox cases were reported in the city of Chicago, and a total of 99 mpox-related communications were posted by the CDPH. The number of weekly reported mpox cases ranged from 0 to 143, with an average of 21.75 cases (SD 38.04). The number of weekly mpox-related communications ranged from 0 to 20, with an average of 1.90 communications (SD 3.96). A total of 1121/1131 (99.1%) of the reported cases and 97/99 (98%) of the communications occurred during the local epidemic (ie, weeks 1‐43). A moderate, positive correlation existed between aligned weekly reported mpox cases and mpox-related communications (ρ=0.67; see Figure 4); this pattern remained when mpox-related communications were lagged by 1 week (ρ=0.66) and by 2 weeks (ρ=0.65). All correlations were statistically significant (*P*<.001).

**Figure 5.**
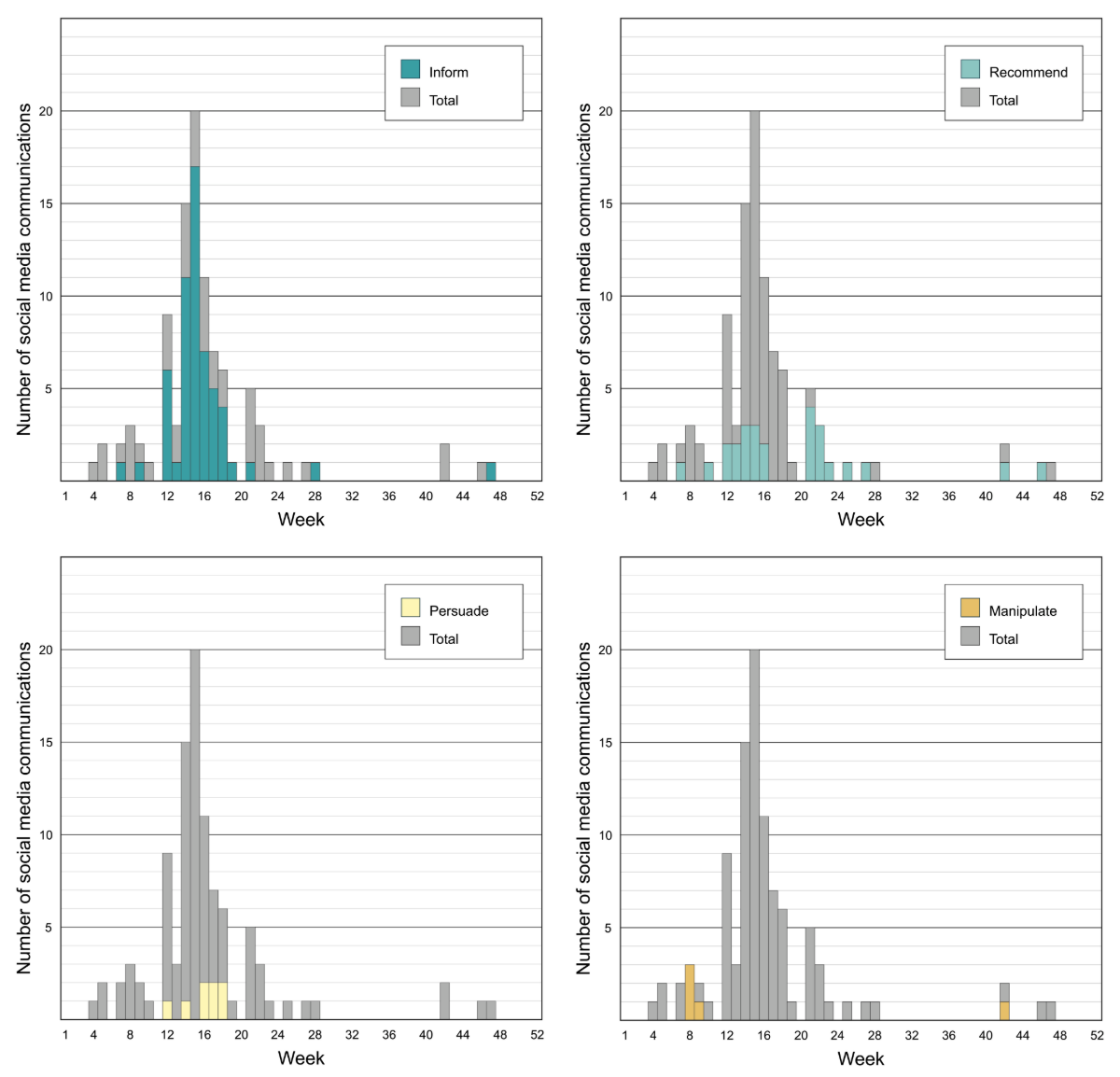
Temporal distribution of the intent of mpox-related communications by the Chicago Department of Public Health.

The schema used to codify the intent of mpox-related communications was robust. The coders agreed on 81 of the 99 communications, yielding an 81.8% agreement rate and a Cohen kappa of 0.63, which is indicative of moderate levels of agreement. In total, 60 (60.6%) of the 99 mpox-related communications sought to inform the public, and 33 sought to influence behavior—with 26 (26.3%) recommending behavior change, 8 (8.1%) seeking to persuade the public to change behavior, and 5 (5%) seeking to manipulate the public to change behavior (see Figure 5). None of the communications were coercive in nature.

## Discussion

### Principal Results

This research examined original communications posted to X by the CDPH to conduct a cross-sectional content analysis that investigated what information was presented, why health officials were communicating, the nature of communications, and the impact of select communication attributes on public engagement.

Results demonstrate that communicable diseases were the most frequently discussed topic, that most communications were posted in English, during the standard workday and with additional media, and that all communications were proactive in nature. Just over half of the communications posted by the CDPH discussed communicable diseases. The next most frequently discussed topics were other health topics and mental health. Other health topics representing the second most frequently discussed topic is the result of the coding schema used in this research. The other health topics category included the general public health subtopic, which included communications discussing interactive online social media forums (eg, Twitter Live). More granularly, 173 of the 245 (70.6%) of the communications coded as other health topics involved these forums. The forums often involved live question-and-answer sessions with CDPH officials on a variety of health-related topics. This strategy involving live social media forums is one previously championed by the CDPH. For example, in 2013, the CDPH conducted X-based communication campaigns seeking to discuss seasonal influenza with Chicagoans using live chats with the acting CDPH Commissioner [26].

It is reasonable to predict that most communications would have been posted, or scheduled to be posted, before 9 AM or after 4:59 PM—when most individuals are not working and may be more likely to use social media platforms—in efforts to maximize reach and audience engagement. Still, over 90 percent of the communications were posted during the standard workday, and 9 AM to 9:59 AM, 11 AM to 11:59 AM, and 12:00 PM to 12:59 PM represent the times that communications were most often posted. One of the benefits of social media, however, is that audiences are not beholden to broadcasting schedules, and individuals can seek information at their convenience. For this reason, the CDPH may have determined that communicating outside of the workday did not hold meaningful implications for public engagement. Results from the regression analysis support this stance, as communications posted from 9 AM to 4:59 PM received significantly higher views compared to communications posted outside this window.

In addition, while social media platforms can improve risk communication, they can also represent conduits for the rapid dissemination of misinformation, disinformation, and malinformation, particularly in the early stages of an emergency [12,16,27,28]. Indeed, as Moore et al [16] have written, “when Jonathan Swift wrote, ‘falsehood flies, and the truth comes limping after it,’ in 1710 he could easily have been describing the state of social media use for public health in [the present day].” Results from this study demonstrate the CDPH used X to exclusively engage in 1-way communication during the study period and did not engage in 2-way dialogue or to reply to misinformation and disinformation. Other research efforts have produced similar results and found local and state health departments most often use social media as a channel to distribute information rather than engage the public and create conversation [13,29]. Recent research has also suggested that the COVID-19 pandemic resulted in a communication strategy shift for public health departments that was characterized by an increase in communication volume but a decrease in interactive engagement [30]

Some evidence suggests that 1-way communication strategies can play an important role in public health response efforts [5,31]. However, 2-way communication is the essence of social media [10], and numerous researchers who have investigated the infodemics accompanying the COVID-19 pandemic and mpox epidemic have concluded that public health authorities offering accurate and reliable information via social media platforms could represent a method for countering the spread of misinformation [32-37]. Failing to engage in more conversational communication appears to be akin to failing to use social media to effectively combat the infodemics that accompanied these emergencies. This is especially true when considering the use of directed mentions and message replies is thought to promote public trust levels, and messaging from official sources of information, such as local health authorities, appears to have an inoculative effect against misinformation and disinformation circulating on social media [38-40].

Still, it may be that the CDPH was responding to misinformation and disinformation, albeit not directly. Others have suggested that direct responses could narrow the audience of communications and that public communications for general audiences help maximize the reach of a message [41]. Thus, many of the CDPH communications may have been motivated by a recognized need to respond to incorrect information circulating online but disseminated in a way believed to maximize reach. Other LHDs in the United States embraced similar approaches. For example, in Baltimore, officials developed a social media-based campaign to combat circulating misinformation and address behaviors related to preventative measures for COVID-19 [42]. Additional research is needed to explore the underlying motivations and implementation of social media communication strategies used by LHDs in the United States.

Findings from this study also suggested that communications with additional media received significantly higher levels of engagement compared to communications without media. This finding aligns with the results obtained by others that used retransmission (ie, “retweeting”) to quantify engagement [41,43]. Recognizing this, in the future, health officials may wish to include images or videos with communications that they hope will receive heightened public engagement. Critically, this strategy should be used judiciously, as it seems unlikely that communications containing additional media will continue to enjoy heightened attention if all posted communications contain media.

Results also suggest that—although this study was conducted during 2 public health emergencies—communications related to those topics were not top of mind for the audience. Instead, communications primarily discussing maternal and child health, mental health, and substance use received significantly higher levels of public engagement, while notably, communications discussing communicable diseases did not. This underscores a need to develop strategies focused on retaining and recapturing the public’s attention when it wanes due to “pandemic fatigue” during prolonged public health emergencies [44,45].

Finally, it is notable that CDPH communications on X were only posted in English and Spanish. Previous experiences suggest that appropriately translating and communicating health information is a resource-intensive endeavor that presents challenges for many LHDs, especially during responses to public health emergencies [46]. However, other LHDs servicing large cities in the United States posted messages to X in a more diverse suite of languages. For example, the Houston Health Department posted the same message on X in Arabic, Chinese, English, Spanish, and Vietnamese (see Multimedia Appendix 2). Furthermore, Chicago is home to an estimated 900,000 individuals who speak a language other than English, including some 368,000 who speak English “less than well” [47]. While Spanish represents the second most spoken language in the city—with an estimated 603,000 Chicagoans speaking Spanish—some 154,000 inhabitants speak another Indo-European language and an additional 107,000 speak an Asian and Pacific Island language [47]. Considering this, it is then somewhat surprising that none of the communications included languages other than English and Spanish—especially when acknowledging the importance of message accessibility and the city’s linguistic diversity.

This noted, it is important to acknowledge that results from the regression analysis suggest that CPDH communications posted in Spanish received significantly lower levels of public engagement. It is difficult to discern if this finding is reflective of inherent or learned health information-seeking preferences. Put another way, it remains unknown if this result is due to the inherent information-seeking preferences of Hispanic communities in Chicago, or if these individuals have learned to seek health information elsewhere because of the CDPH practices that heavily favor communications in the English language. Irrespective of the cause, these results underscore the importance of developing strategies for conducting outreach with communities that communicate in languages other than English to provide them with information during public health emergencies, as well as a need for additional, practice-oriented research focused on information-seeking preferences of various ethnic groups.

### Mpox-Related Results

The mpox subanalysis examined whether the number of mpox-related communications was correlated with the number of reported cases and the intent of mpox-related messaging. Results demonstrate that moderate, positive correlations existed between mpox-related communications and reported mpox cases and that a majority of the CDPH communications related to mpox sought to inform the public.

The most notable result from the correlational analysis was that—while all correlations were positive, of moderate strength, and statistically significant—the strongest correlation existed when communications and cases were aligned temporally. Others have discussed how much of the discussions surrounding mpox on X were largely misinformed and predated accurate health messaging from public health officials [35]. Indeed, other research has demonstrated that many public communications posted from May to July 2022 discussed mpox related to conspiracy theories about the existence or origins of the outbreak, as well as homophobic and racial comments [48,49]. This highlights the importance of accurate, fact-based messaging on social media by public health officials early during infectious disease outbreaks. While it is reasonable to expect the number of mpox-related communications to have increased following increases in reported cases, which would have resulted in stronger correlations in the lagged analyses, this does not appear to have been the case in Chicago during the mpox epidemic. The CDPH should, therefore, be commended in its apparent efforts to provide accurate information about mpox during a critical period of time in the outbreak response.

Regarding the intent of mpox-related messaging, others have characterized effective outbreak risk communication messaging as that which informs the public, ultimately as a means of motivating appropriate self-protective behaviors [45,50]. Results from this research suggest that most mpox-related communications sought to inform the public and did not explicitly seek to influence behavior. Most of the informative communications posted by the CDPH provided information about the number of local mpox cases, vaccine eligibility and availability, and other resources. Relatively few of the informative communications discussed mpox symptoms, as these messages often came with calls to action (eg, “contact your healthcare provider”). Because of this, these communications were coded as intending to recommend behavior change or persuade the public to modify behaviors. These results align with those from previous research that found that LHDs using X to communicate about Ebola were mostly using the platform to inform the public [31].

Of the mpox-related communications that did seek to influence behavior, most recommended behavior change or sought to persuade the public through appeals to logic and reason; only 5 of the communications were found to have manipulative intentions, and none of the communications were coercive. Most of the manipulative communications occurred relatively early in the outbreak because of pride events that sought to celebrate the lesbian, gay, bisexual, transgender, queer, and intersex (LGBTQI+) communities. The manipulative communications posted by the CDPH appealed to the emotions of these demographic groups using messages like, “pride is greater than fear” or, “don’t let anything stand in the way of your pride.”

While this messaging demonstrates an awareness of epidemiological trends and targeted messaging—as these populations bore a disproportionately large burden of disease during the epidemic [51,52]—it is also important to recognize how limitations related to social media platforms could have presented challenges for conveying nuanced information. For example, research by Shi et al [53] found that mpox-related information on another popular social media platform, TikTok, is generally of poor quality and lacking the content and context to provide accurate and comprehensive knowledge. Similar concerns exist with X because of the character limits imposed on communications, which may have complicated discussions surrounding the disease. For example, ideally, communications would have distinguished mpox as a sexually transmissible disease and not a sexually transmitted disease, but character limits could have presented challenges to this objective. In the case of the CDPH, the communications that targeted LGBTQI+ populations could have also inadvertently contributed to the incorrect perception that mpox was a “gay disease” that only impacted certain populations.

These limitations associated with social media-based communications and their implications for stigmatization cannot be discounted, as there is a rich history of stigmatization during infectious disease epidemics. While the stigma faced by the LGBTQI+ community throughout the HIV/AIDS outbreak represents the most infamous example, other notable examples include stigmatizing Asian communities during the SARS epidemic in 2003 and COVID-19 in 2020, Latinx communities during the 2009 H1N1 influenza pandemic, and African communities during recent Ebola outbreaks [54]. This stigmatization can hold serious consequences for implementing an effective outbreak response; it can lead infected persons to misreport symptoms and delay seeking health care when needed, result in elevated stress and depressive symptoms in stigmatized groups, and contribute to the politicization of outbreak responses [52,54-56]. Ultimately, because of this, it is reasonable to question if the use of certain social media platforms, including X, during outbreak response should be limited to certain functions, such as providing epidemiologic updates or directing the public to more comprehensive resources. It also supports the position that social media should not be viewed as a strategy in and of itself for risk communication, but rather as a tool that can be used to supplement other information sources and outreach efforts [15,28,57].

### Limitations

This research has several limitations. First, while the text contained in the image-based media accompanying communications was considered in this research, the content of video-based media was not. The information provided in these videos could have impacted the coding of these communications, though it is difficult to discern the potential impact on research results.

Second, an inherent challenge to conducting research involving social media platforms, including X, is accounting for the impact of dynamic algorithms. Previous research has used retransmission as a proxy measure for public engagement [41,43]. However, using view counts was not an option when these studies were conducted, and this research elected to use view counts to quantify public engagement because it represents a more direct measure of public engagement. Still, because of the novelty of view counts, methods for addressing the bias introduced by algorithms have not yet been developed. Because of this, it is difficult to control for the bias that may be introduced by algorithms or hypothesize how they may impact the results.

Third, this study did not assess the causal effects of communications on public behavior. This is important, as systematic reviews have demonstrated that the effects of health communication campaigns can vary significantly according to their focus [58,59]; other factors like politics can also influence public behavior and the response to risk communication efforts [60]. While critical to note, this consideration falls beyond the scope of this research, and future work may wish to investigate how communications by public health officials influenced behavior during these public health emergencies.

Finally, the results from this research have limited external validity. This research represents a cross-sectional case study investigating the communications of a single LHD, using a single social media platform, in a large American city, during a unique time period. Because of this, these results should not be considered as representative of the communication practices of other LHDs, of the practices on other social media platforms, or during other periods of time. Additional comparative research should consider how the results presented here may differ when considering different cities, different social media platforms, and different contexts.

### Conclusions

This study found that over the course of a year, a majority of the CDPH communications on X discussed communicable diseases, were posted in English during the standard workday, and the CDPH used X for proactive communication. Regression analysis demonstrated that communications with media, those posted during the standard workday, and those discussing maternal and child health, mental health, and substance use received significantly higher levels of engagement, while communications posted in Spanish received significantly lower levels of engagement. Results also demonstrated that the weekly mpox-related communications were positively correlated with weekly reported mpox cases at a moderate and statistically significant level, and that most mpox-related communications were posted with the intent of informing the public and did not explicitly attempt to influence behavior.

These results underscore the need for a multifaceted and multipronged approach for risk communication during public health emergencies. While social media platforms can represent valuable, low-cost methods for communication, they should supplement other vehicles for dissemination that may be more appropriate for communicating certain types of information, achieving behavior change, or reaching certain communities and demographic groups. Ideally, health officials would know which audiences typically engage with their social media as a means of tailoring these efforts, while also working to ensure that posted communications are clear, accessible in a variety of languages, use media to boost public engagement, and direct the public to more comprehensive resources for communicating nuanced information when necessary.

## Supplementary material

10.2196/68200 Multimedia Appendix 1 Health topic coding schema.

10.2196/68200 Multimedia Appendix 2 Houston Health Department’s multilingual social media postings.

## Abbreviations

CDPH: Chicago Department of Public Health
ERR: event rate ratio
LGBTQI+: lesbian, gay, bisexual, transgender, queer, and intersex
LHD: local health department
