# Supplementary material for: Chicago Public Health Department Social Media Communications on Twitter During the COVID-19 Pandemic and the Mpox Epidemic: Cross-Sectional Content Analysis
Source: J Med Internet Res. 2025 Jul 18;27:e68200. doi: 10.2196/68200 (PMC12294644; doi:10.2196/68200)
Supplement: Multimedia Appendix 1 [file jmir-v27-e68200-s001.docx]

# Appendix 1. Health topic coding schema.

| **Primary Health Topic** | **Secondary Health Topic(s)** |
| --- | --- |
| 1. Communicable disease | 1.1. Acute respiratory illness |
|  | 1.2. Antibiotics & antimicrobial resistance |
|  | 1.3. COVID-19 |
|  | 1.4. Food-borne disease |
|  | 1.5. Fungal infections |
|  | 1.6. HIV / Sexually Transmitted Infections |
|  | 1.7. Mpox |
|  | 1.8. Routine vaccine preventable disease |
|  | 1.9. Multiple communicable diseases |
| 2. Environmental health | 2.1. Built environment |
|  | 2.2. Green space |
| 3. Maternal and child health | 3.1. Infant and newborn health |
|  | 3.2. Reproductive health |
| 4. Mental health |  |
| 5. Noncommunicable disease | 5.1. Cancer |
|  | 5.2. Cardiovascular disease |
|  | 5.3. Diabetes |
|  | 5.4. Multiple noncommunicable diseases |
| 6. Public safety and violence | 6.1. Domestic violence |
|  | 6.2. Gun violence |
|  | 6.3. Human trafficking |
|  | 6.4. Public safety |
| 7. Substance use | 7.1. Controlled substance use |
|  | 7.2. Tobacco / nicotine product use |
| 8. Multiple health topics |  |
| 9. Other, health topics | 9.1. CPR |
|  | 9.2. Emergency preparedness |
|  | 9.3. Gender |
|  | 9.4. General public health |
|  | 9.5. Healthcare access and insurance |
|  | 9.6. Health equity and inclusion |
|  | 9.7. Hypothermia / frostbite |
|  | 9.8. Neurodevelopmental disorders |
|  | 9.9 Nutrition |
|  |  |
| 10. Other, non-health topics |  |
