# Supplementary material for: Chicago Public Health Department Social Media Communications on Twitter During the COVID-19 Pandemic and the Mpox Epidemic: Cross-Sectional Content Analysis
Source: J Med Internet Res. 2025 Jul 18;27:e68200. doi: 10.2196/68200 (PMC12294644; doi:10.2196/68200)
Supplement: Multimedia Appendix 2 [file jmir-v27-e68200-s002.docx]

**Appendix 2. Houston Health Department’s multilingual (Arabic, Chinese, English, Spanish, Vietnamese) social media postings.**


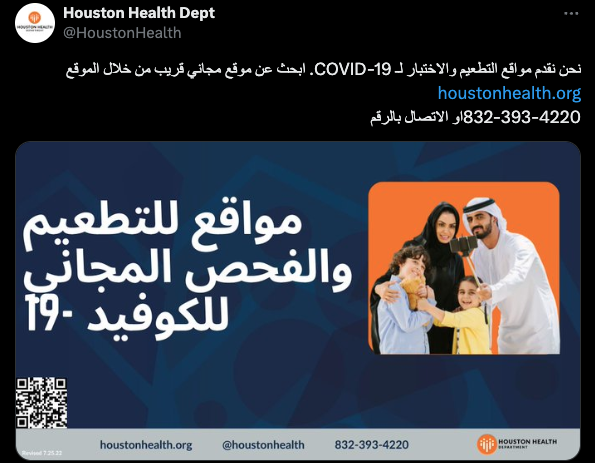

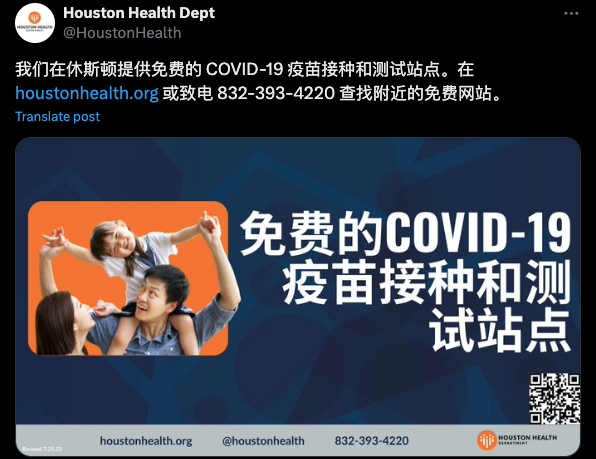


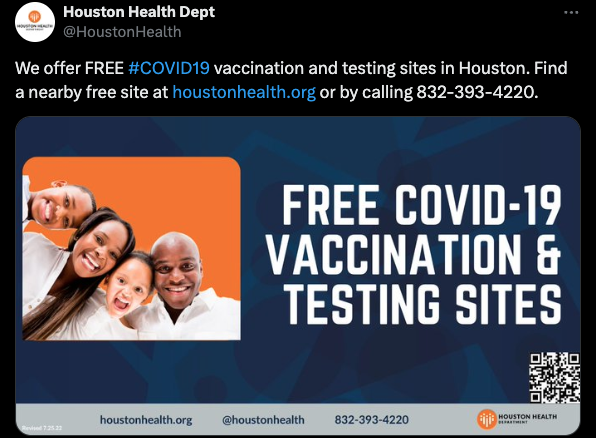
 **
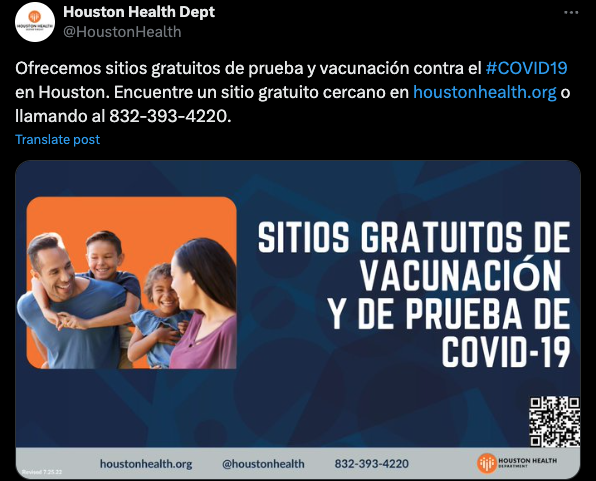
**


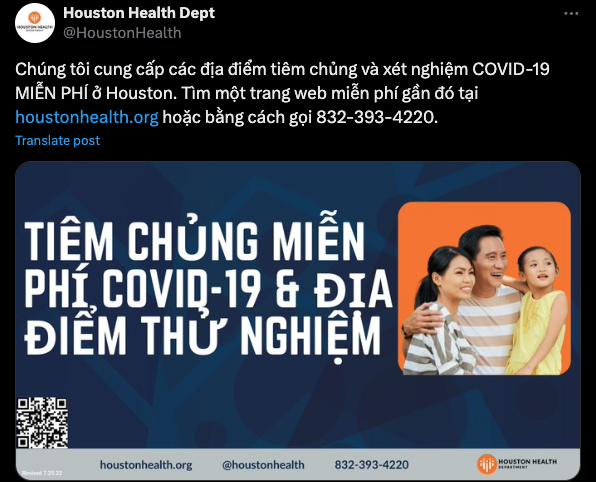


Link to Arabic communication: <https://x.com/HoustonHealth/status/1752723702242841058>

Link to Chinese communication: <https://x.com/HoustonHealth/status/1752406663577956709>

Link to English communication: <https://x.com/HoustonHealth/status/1751989905985519745>

Link to Spanish communication: <https://x.com/HoustonHealth/status/1752361759560663113>

Link to Vietnamese communication: <https://x.com/HoustonHealth/status/1752044346218610764>
